# Effects of endotoxin adsorber hemoperfusion on sublingual microcirculation in patients with septic shock: a randomized controlled trial

**DOI:** 10.1186/s13613-020-00699-z

**Published:** 2020-06-12

**Authors:** Shih-Hong Chen, Wing-Sum Chan, Chih-Min Liu, Ching-Tang Chiu, Anne Chao, Vin-Cent Wu, Wang-Huei Sheng, Chien-Heng Lai, Ming-Jiuh Wang, Yu-Chang Yeh

**Affiliations:** 1 Department of Anesthesiology, Taipei Tzu Chi Hospital, No. 289, Jianguo Rd., New Taipei, Taiwan; 2 Institute of Molecular Medicine, National Tsing Hua University, No. 101, Section 2, Kuang-Fu Road, Hsinchu, Taiwan; 3 Department of Anesthesiology, Far Eastern Memorial Hospital, No. 21, Sec. 2, Nanya S. Rd., New Taipei, Taiwan; 4 Department of Anesthesiology, National Taiwan University Hospital, No 7, Chung Shang South Road, Taipei, Taiwan; 5 Department of Internal Medicine, National Taiwan University Hospital, No 7, Chung Shang South Road, Taipei, Taiwan; 6 Department of Surgery, National Taiwan University Hospital, No 7, Chung Shang South Road, Taipei, Taiwan

**Keywords:** Endotoxin, Microcirculation, Septic shock

## Abstract

**Background:**

Endotoxins can induce an excessive inflammatory response and result in microcirculatory dysfunction. Polymyxin-B hemoperfusion (PMX-HP) has been recognized to effectively remove endotoxins in patients with sepsis and septic shock, and a rat sepsis model revealed that PMX-HP treatment can maintain a better microcirculation. The primary aim of this study was to investigate the effect of PMX-HP on microcirculation in patients with septic shock.

**Methods:**

Patients with septic shock were enrolled and randomized to control and PMX-HP groups. In the PMX-HP group, patients received the first session of PMX-HP in addition to conventional septic shock management within 24 h after the onset of septic shock; the second session of PMX-HP was provided after another 24 h as needed.

**Results:**

Overall, 28 patients finished the trial and were analyzed. The mean arterial pressure and norepinephrine infusion dose did not differ significantly between the control and PMX-HP groups after PMX-HP treatment. At 48 h after enrollment, total vessel density (TVD) and perfused vessel density (PVD) were higher in the PMX-HP group than in the control group [TVD 24.2 (22.1–24.9) vs. 21.1 (19.9–22.9) mm/mm^2^; *p* = 0.007; PVD 22.9 (20.9–24.9) vs. 20.0 (18.9–21.6) mm/mm^2^, *p* = 0.008].

**Conclusions:**

This preliminary study observed that PMX-HP treatment improved microcirculation but not clinical outcomes in patients with septic shock at a low risk of mortality. Nevertheless, larger multicenter trials are needed to confirm the effect of PMX-HP treatment on microcirculation in patients with septic shock at intermediate- and high-risk of mortality.

*Trial registration* ClinicalTrials.gov protocol registration ID: NCT01756755. Date of registration: December 27, 2012. First enrollment: October 6, 2013. https://clinicaltrials.gov/ct2/show/NCT01756755

Severe microcirculatory dysfunction is associated with multiple organ injury and mortality in patients with septic shock [1–3]. Microcirculatory dysfunction includes endothelial damage, impaired vasoregulation, and coagulation activation [4, 5], and this dysfunction may present as capillary leakage, hypotension, microthrombosis and impair the tissue perfusion. One of the leading causes of microcirculatory dysfunction is endotoxin, which can induce excessive immune reactions, inflammatory responses, and oxidative stress [6]. Endotoxin injury can be reduced by antagonization or removal strategy. A Toll-like receptor 4 antagonist was reported to improve microcirculation in endotoxemic rats [7]. Moreover, direct hemoperfusion with a polymyxin B-immobilized column was determined to be effective to reduce circulating endotoxins [8]. A rat sepsis model revealed that removal of circulating endotoxin using polymyxin-B hemoperfusion (PMX-HP) can maintain a better microcirculation and lower damage markers [9]. Notably, poor microcirculation parameters reflect inadequate tissue perfusion [10, 11]; thus, the improvement of microcirculation may ensure adequate tissue perfusion and prevent ischemic damage of organs. To the best of our knowledge, no clinical study has investigated the effect of PMX-HP treatment on microcirculation in patients with septic shock. Therefore, we hypothesized that PMX-HP treatment can improve microcirculation by removing endotoxin and reducing endotoxin-related microcirculatory dysfunction. The primary aim of this study was to investigate the effects of PMX-HP on microcirculation in patients with septic shock.

## Methods

### Study design and patient selection

This prospective, randomized, controlled study was approved by the Research Ethics Committee of National Taiwan University Hospital (approval number: 201208067RIB) and registered on the ClinicalTrials.gov protocol registration system (ID: NCT01756755). This study was conducted between October 2013 and July 2018. The definition of sepsis and septic shock met the criteria of international consensus definition [12, 13]. Inclusion criteria for patients with septic shock were intra-abdominal infection with adequate management, proven gram-negative bacteria infection, or endotoxin activity assay (EAA) level of > 0.6 EAA units in patients with pneumonia, blood stream infection, or urinary tract infection. Exclusion criteria were age less than 20 years, the onset of sepsis and septic shock more than 24 h at enrollment, pregnancy, participation in interventional trials at other intensive care units (ICUs) within 30 days before enrollment, undergoing organ transplant surgery within 1 year before enrollment, life-expectancy less than 30 days, history of cardiopulmonary resuscitation (CPR) within 30 days before enrollment, signed no-CPR consent before enrollment, hemophilia, allergic history to polymyxin B or heparin, uncontrolled bleeding within 24 h before enrollment, renal replacement therapy before enrollment, white blood cells count less than 0.5 K/uL or platelet count less than 50 K/uL, human immunodeficiency virus infection, Acute Physiology and Chronic Health Evaluation (APACHE) II score higher than 30 at enrollment, and non-native speakers. Moreover, patients were not enrolled if they declined to participate. Informed consent was obtained from patients’ legally authorized representatives before enrollment. After enrollment, patients were randomly assigned to the control and PMX-HP groups based on the opaque, sealed envelope technique. In the control group, septic shock was treated according to the practice guidelines for sepsis and septic shock [13, 14]. In the PMX-HP group, patients received one session of PMX-HP within 24 h after the onset of septic shock in addition to conventional septic shock management. Sublingual microcirculation video sequences were recorded using a sidestream dark field video microscope (MicroScan; Microvision Medical, Netherlands) at the following time points: T0, enrollment; T1, 24–26 h after T0; and T2, 48 h after T0. At T1, patients in the PMX-HP group received a second session of PMX-HP if the patient’s septic shock was not resolved. At each time point, clinical data, mean arterial pressure (MAP), norepinephrine infusion dose, APACHE II score, sequential organ failure assessment (SOFA) score, laboratory data, the length of ICU and hospital stay, and survival status at 28 days were recorded. Arterial oxygen tension/fraction of inspired oxygen concentration (PaO_2_/FiO_2_) ratio was recorded if the data were available.

### PMX-HP treatment protocol

PMX-HP was performed using an extracorporeal hemoperfusion cartridge with polymyxin B immobilized on polystyrene fibers (Toraymyxin PMX-20R, Toray Industries, Tokyo, Japan). Cartridge and circuit were first washed using 4 L 0.9% saline and then primed with 4000 IU heparin in 1 L 0.9% saline. Vascular access was obtained using double-lumen venous catheter. The blood was perfused at a flow rate of 100 to 150 mL/min for 2 h. During PMX-HP treatment, patients received heparin at a loading dose of 3000 IU and a maintenance dose of 20 U/kg/h following manufacturer’s instruction. Notably, the heparin dose was adjusted according to our heparin dosing score protocol in patients with coagulopathy to avoid any bleeding event [15].

### Measurements of sublingual microcirculation

Five video sequences (length: 20 s each) were recorded at different sites on ventral aspect of the tongue according to the consensus guidelines [16] by one of the two operators, a clinical research nurse (Ms. Wang) and Dr. Yeh, who had been trained and taken more than 300 and 100, respectively, microcirculation recordings for patients and health volunteers. These video sequences were digitally stored with code numbers to ensure the anonymity of patient information. Subsequent microcirculation analyses were performed according to the consensus guidelines [16] by a research assistant (Ms. Wu, who had been trained and analyzed more than 3000 video sequences of animal and human microcirculation) who was blinded to the patient information. Three sequences with appropriate image quality were selected for analysis using the semi-automated analysis software Automated Vascular Analysis 3.0 [17]. Inappropriate image quality included pressure or secretion artifact, and inadequate focus and contrast adjustments [17]. The following parameters were investigated: (a) total vessel density (TVD; vessels less than 20 μm), (b) perfused vessel density (PVD), (c) proportion of perfused vessels (PPV), and (d) microvascular flow index (MFI) score. TVD was automatically calculated by the software. The blood flow in small vessels was classified using an ordinal scale of 0–3, and small vessels with a blood flow classification of 2 or 3 were considered as perfused vessels [18]. PVD was semi-automatically calculated by the software. The MFI scores were semiquantitatively calculated according to suggestions made at the roundtable conference [19].

### End points and sample size analysis

The primary end point was the difference in PVD between the control and PMX-HP groups at T2. Based on our experience, 20 patients per group were sufficient to detect a 12% difference of PVD between the two groups, with an α level of 0.05 (two-tailed) and a β level of 0.2, assuming a controlled mean PVD of 20.0 mm/mm^2^ with a standard deviation of 3.0. The secondary end points included the difference in APACHE II score, SOFA score, and MAP between the two groups at T2.

### Statistical analysis

All statistical analyses were performed using SPSS version 20 (IBM, Armonk, NY, USA). Normally distributed numerical data were expressed as means (standard deviation) and compared using *t* test. Non-normal distributed numerical data, TVD, and PVD were expressed as medians (interquartile range) and compared using the Mann–Whitney test. Categorical variables were described as percentages and were compared using the Chi-square test or Fisher’s exact test as appropriate. Intention-to-treat analysis was used for most comparisons between the two groups. Intention-to-treat, as-treated, and per-protocol analysis were used to investigate the difference in PVD between the two groups. A *p* value of < 0.05 was considered statistically significant.

## Results

### Patient characteristics

**Fig. 1:**
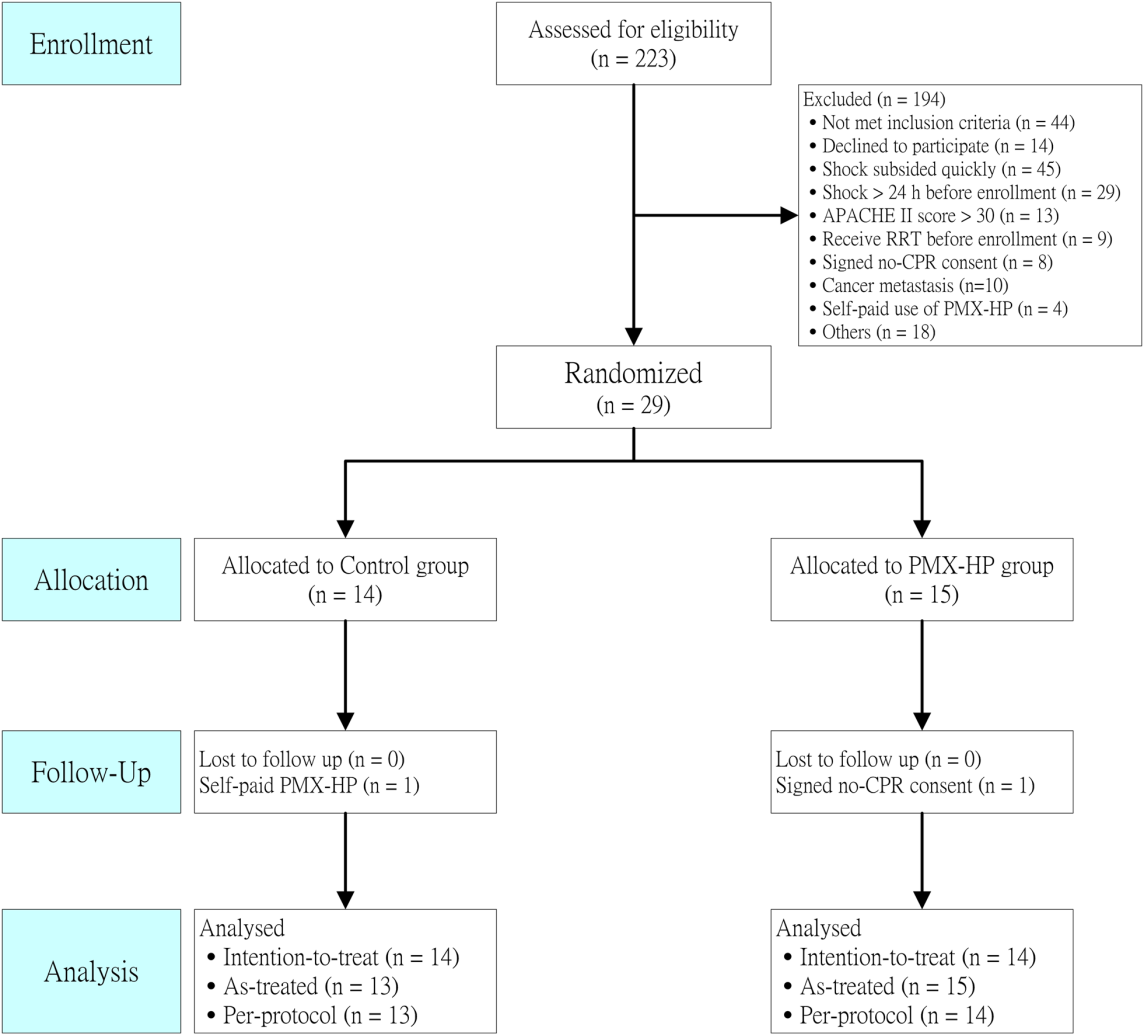
Consort flowchart of patient enrollment. *APACHE* acute physiology and chronic health evaluation, *CPR* cardiopulmonary resuscitation, *PMX*-*HP* polymyxin B hemoperfusion

A total of 223 patients with severe sepsis and septic shock were initially considered for inclusion in this trial (Fig. 1). Subsequently, 194 patients were excluded, and 29 patients were randomized. However, in the PMX-HP group, one patient signed no CPR consent and decided to pursue palliative care after enrollment. Therefore, finally, 28 patients were analyzed. In the PMX-HP group, 10 patients received one session of PMX-HP, and 4 patients received two sessions of PMX-HP. In the control group, one patient requested self-financed PMX-HP and received one session of PMX-HP. Patient characteristics are listed in Table 1. Patients’ characteristics did not differ significantly between the control and PMX-HP groups.

**Table 1:** Patient characteristics, hemodynamic and laboratory data, and treatments for septic shock

Group	Control	PMX-HP	*p* values
Number of patients	14	14	
Age (in years)	68.2 (14.3)	67.7 (10.8)	0.920
Female, *n* (%)	7 (50%)	8 (57%)	1.000
Height (cm)	158 (9)	162 (13)	0.368
Weight (kg)	62.7 (12.2)	64.7 (17.5)	0.723
APACHE II score	19 (6)	19 (5)	0.973
At T0			
SOFA score	9 (3)	9 (2)	0.890
Intra-abdominal infection, *n* (%)	13 (93%)	13 (93%)	1.000
WBC count (k/μL)	12.5 (5.2–37.6)	14.9 (6.5–27.5)	0.603
Hemoglobin (g/dL)	11.1 (1.7)	12.0 (2.4)	0.292
Body temperature (°C)	37.3 (1.0)	38.0 (1.0)	0.071
Heart rate (bpm)	109 (22)	111 (17)	0.843
MAP (mmHg)	76 (10)	75 (7)	0.709
Lactate (mmol/L)	4.1 (3.4)	4.0 (3.3)	0.940
Norepinephrine (mcg/kg/min)	0.11 (0.05–0.17)	0.20 (0.13–0.28)	0.056
At T1			
SOFA score	7 (4)	7 (4)	0.919
WBC count (k/μL)	18.1 (7.1–35.8)	16.3 (9.8–20.9)	0.344
Hemoglobin (g/dL)	9.5 (1.5)	9.7 (1.5)	0.749
Body temperature (°C)	36.7 (1.0)	36.9 (0.6)	0.434
Heart rate (bpm)	90 (10)	96 (21)	0.346
MAP (mmHg)	84 (11)	83 (11)	0.764
Lactate (mmol/L)	2.5 (1.4)	2.3 (1.2)	0.688
Norepinephrine (mcg/kg/min)	0.01 (0–0.03)	0.01 (0–0.06)	0.511
Fluid supplement (0–24 h) (mL)	3639 (3056–4177)	3811 (2652–4908)	0.874
At T2			
SOFA score	6 (4)	6 (4)	0.877
WBC count (k/μL)	14.6 (12.0–21.3)	17.6 (13.6–22.3)	0.525
Hemoglobin (g/dL)	9.8 (1.2)	9.4 (1.3)	0.503
Body temperature (°C)	36.6 (0.7)	37.1 (1.1)	0.157
Heart rate (bpm)	90 (19)	87 (19)	0.647
MAP (mm Hg)	86 (13)	83 (13)	0.518
Lactate (mmol/L)	1.4 (0.8)	1.8 (0.8)	0.354
Norepinephrine (mcg/kg/min)	0 (0–0)	0 (0–0.01)	0.657
Fluid supplement (25–48 h) (mL)	2452 (1762–3101)	2339 (1773–3190)	1.000

Data are presented as the mean (SD) for normal distribution data or median (interquartile range) for non-normal distribution data

*APACHE* acute physiology and chronic health evaluation, *MAP* mean arterial pressure, *PMX*-*HP* polymyxin B hemoperfusion, *SOFA* sequential organ failure assessment, *WBC* white blood cells

### Hemodynamic parameters, laboratory data, and clinical outcomes

Patients’ hemodynamic data, laboratory data, and treatments for septic shock are listed in Table 1. MAP and norepinephrine infusion dose did not differ significantly between the control and PMX-HP groups at T1 and T2. Only one patient in the PMX-HP group required an additional infusion of epinephrine at enrollment, but the infusion was discontinued 4 h after PMX-HP treatment. Total fluid supplementation within the first 48 h did not differ significantly between the PMX-HP and control groups [6025 (4690–7623) vs. 6034 (5012–7247) mL, *p* = 0.946]. No significant intergroup differences were noted regarding changes in the SOFA score and APACHE II score from T0 to T2. The total urine output over 48 h after enrollment did not differ significantly between the PMX-HP and control groups [3615 (2170–5075) vs. 3365 (2373–5078) mL, *p* = 0.946]. The creatinine level at T1 was nonsignificantly lower in the PMX-HP group than in the control group [1.1 (0.6) vs. 1.9 (1.5) mg/dL, *p* = 0.110]. The platelet counts did not differ significantly between the PMX-HP and control groups at T1 and T2 [T1, 134 (63) vs. 162 (141) k/μL, *p* = 0.504; T2, 122 (47) vs. 127 (83) k/μL, *p* = 0.880]. PaO_2_/FiO_2_ ratio did not differ significantly between the PMX-HP and control groups [T0, *n* = 14 vs 14, 244 (125) vs. 265 (147), *p* = 0.689; T1, *n* = 10 vs. 13: 346 (133) vs. 346 (116), *p* = 0.999; T2, *n* = 8 vs. 6: 342 (167) vs. 285 (117), *p* = 0.495].

Patients’ clinical outcomes and survival are presented in Table 2. No significant intergroup differences were noted regarding the ICU stay, hospital stay, and 28-day survival.

**Table 2:** Patients’ outcomes and survival

Group	Control	PMX-HP	*p* values
Number of patients	14	14	
Renal replacement therapy, *n* (%)	2 (14%)	3 (21%)	1.000
Ventilator use (days)	5 (3–8)	5 (3–13)	0.571
Intensive care unit stay (days)	7 (5–12)	9 (5–20)	0.685
Hospital stay (days)	22 (15–50)	29 (19–51)	0.479
28-day survival, *n* (%)	13 (93%)	13 (93%)	1.000

Data are presented as the number (%) or median (interquartile range)

*PMX*-*HP* polymyxin B hemoperfusion

### Microcirculation parameters

**Fig. 2:**
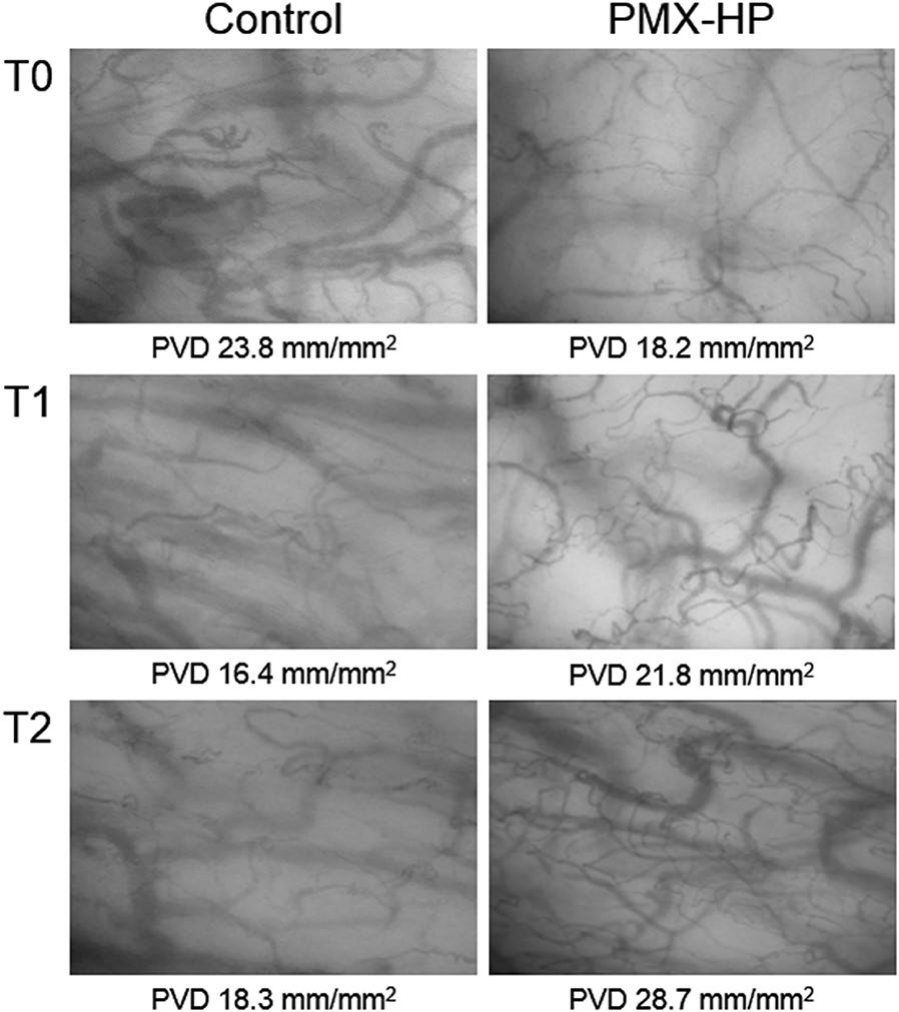
Sublingual microcirculation images in patients with sepsis. Time points: T0, at enrollment; T1, at 24–26 h after enrollment; and T2, 48 h after enrollment. *PMX*-*HP* polymyxin B hemoperfusion, *PVD* perfused vessel density

**Fig. 3:**
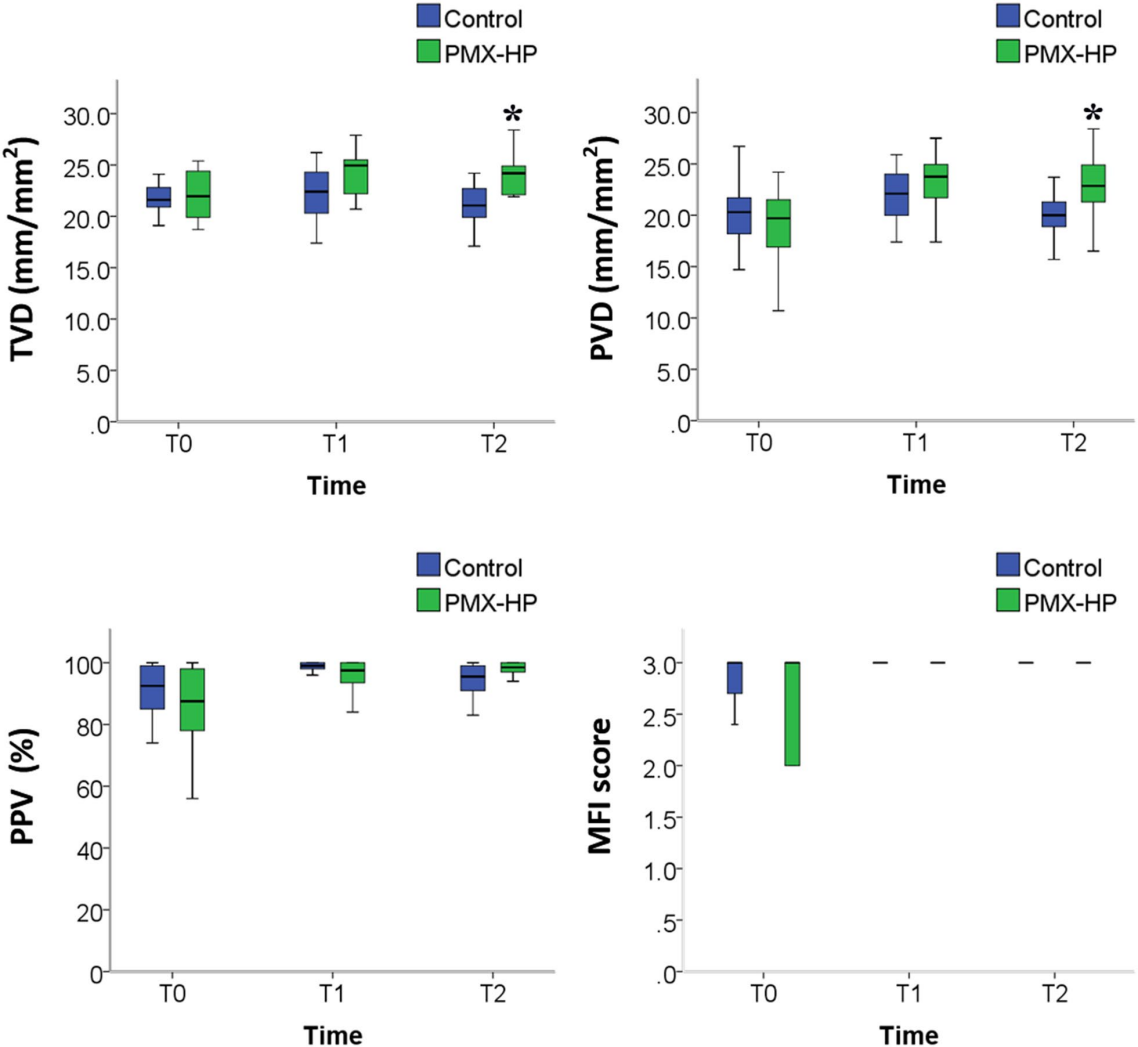
Comparison of microcirculation parameters between the control and PMX-HP groups. *n* = 14 patients in each group. Time points: T0, at enrollment; T1, at 24–26 h after enrollment; and T2, 48 h after enrollment. *MFI* microvascular flow index, *PMX*-*HP* polymyxin B hemoperfusion, *PPV* proportion of perfused vessels, *PVD* perfused vessel density, *TVD* total vessel density. *p < 0.05 by intent-to-treat analysis using Mann–Whitney test

A total of 420 video sequences of sublingual microcirculation were recorded for the 28 enrolled patients, and 252 video sequences with appropriate image quality were analyzed according to the description in Methods. Examples of sublingual microcirculation images are presented in Fig. 2. TVD, PVD, PPV, and MFI of the two groups are presented in Fig. 3. The intent-to-treat analysis at T1 revealed no significant intergroup differences related to TVD and PVD. At T2, TVD and PVD were higher in the PMX-HP group than in the control group [TVD 24.2 (22.1–24.9) vs. 21.1 (19.9–22.9) mm/mm^2^; *p* = 0.007; PVD 22.9 (20.9–24.9) vs. 20.0 (18.9–21.6) mm/mm^2^, *p* = 0.008]. As-treated analysis and per-protocol analysis of TVD and PVD at T1 and T2 are presented in Table 3.

**Table 3:** Different analyses of total vessel density (TVD) and perfused vessel density (PVD)

Group	Control	PMX-HP	*p* values
Intent-to-treat analysis			
Number of patients	14	14	
T1 TVD (mm/mm^2^)	20.8 (20.0–24.1)	23.3 (20.9–25.2)	0.069
T1 PVD (mm/mm^2^)	19.7 (18.8–23.2)	22.8 (20.9–24.5)	0.085
T2 TVD	21.1 (19.9–22.9)	24.2 (22.1–24.9)	0.007
T2 PVD	20.0 (18.9–21.6)	22.9 (20.9–24.9)	0.008
As-treated analysis			
Number of patients	13	15	
T1TVD	20.5 (19.9–23.4)	23.6 (20.9–25.1)	0.041
T1 PVD	19.3 (18.7–22.5)	22.9 (20.9–24.4)	0.037
T2 TVD	20.7 (19.8–22.7)	23.9 (22.1–24.9)	0.003
T2 PVD	19.8 (18.8–21.0)	23.0 (21.3–24.9)	0.002
Per-protocol analysis			
Number of patients	13	14	
T1TVD	20.5 (19.9–23.4)	23.3 (20.9–25.2)	0.048
T1 PVD	19.3 (18.7–22.5)	22.8 (20.9–24.5)	0.048
T2 TVD	20.7 (19.8–22.7)	24.2 (22.1–24.9)	0.005
T2 PVD	19.8 (18.8–21.0)	22.9 (20.9–24.9)	0.004

Data are presented as median (interquartile range)

*PMX*-*HP* polymyxin B hemoperfusion

## Discussion

The question addressed by the present study was whether PMX-HP treatment can improve microcirculation by removing endotoxin and reducing endotoxin-related microcirculatory dysfunction in patients with septic shock. The main finding of this study is that microcirculation in patients with septic shock was improved after PMX-HP treatment. We observed that TVD and PVD were higher in the patients received PMX-HP treatment than in those who received conventional treatment at 48 h after enrollment. However, no significant improvement was observed in the SOFA score, MAP, lactate level, and the total amount of fluid supplementation after PMX-HP treatment.

The finding of improved microcirculation after PMX-HP treatment for septic shock was compatible with the results of the rat sepsis model mentioned in Introduction [9]. Moreover, our previous septic shock pig study revealed that PMX-HP attenuated microcirculatory dysfunction at the ileal mucosa and kidney surface at 6 h after PMX-HP treatment [20]. However, no significant improvement was observed regarding sublingual microcirculation at 6 h after PMX-HP treatment in those pigs with septic shock. This observation indicated that different organ exhibited heterogeneity regarding the timing and severity of microcirculatory dysfunction. Compared with PVD in healthy volunteers in our previous study [3], PVD was 18% (95% confidence interval 10% to 25%) lower at T2 in the control group than in healthy volunteers, and PVD was 7% (95% confidence interval − 1% to 14%) lower, albeit nonsignificantly, at T2 in the PMX-HP group than in healthy volunteers. In addition, the mortality of septic shock in this study was relatively low, and the values of PVD in the two groups were compatible with the values of PVD in survivors with septic shock [3].

This study did not observe significant intergroup differences in the SOFA score, MAP, and lactate level. The reason for the lack of significant clinical benefits after PMX-HP treatment could be the low severity of septic shock with a relatively low mortality rate of 7% in our enrolled patients. The low mortality rate was compatible with the SOFA score prediction of mortality; an initial score of 9 and a 48-h score of 6 reflect mortality of less than 10% [21]. Notably, PMX-HP reduced mortality in septic shock patients with intermediate- (30–60%) and high-risks (> 60%) of mortality [22]. PMX-HP did not reduce mortality in septic shock patients with a low risk of mortality (< 30%) [22, 23]. Therefore, additional studies are required to investigate the effect of PMX-HP on microcirculation in patients with septic shock who have intermediate- and high-risks of mortality.

Our study did not show any adverse effects of PMX-HP treatment. Notably, the incidence of adverse events of PMX-HP treatment has been reported to be very low (< 1%), and the most commonly observed adverse effects of PMX-HP are thrombocytopenia, transient hypotension, and allergic reactions [24]. In our study, no significant intergroup difference was observed regarding the platelet count at T1 and T2. Moreover, PMX-HP was reported to remove inflammatory cells [25], but no significant intergroup difference was noted regarding the white blood cell count at T1 and T2.

Our study has several limitations. First, the study sample size was limited by the strict inclusion and exclusion criteria. Many patients did not meet the inclusion criteria and were excluded based on mild or severe severity of septic shock or a prolonged shock more than 24 h before enrollment. Second, surviving sepsis campaign guidelines have continually improved early resuscitation and survival of patients with septic shock. If patients are recognized early and adequately resuscitated in the emergency department or general ward, they may not need admission to ICUs or require the PMX-HP treatment. Because of the slow progress in recruiting participants after an extended enrollment for more than 4 years, we decided to stop the study before the target sample size was reached. Third, PMX-HP treatment requires heparin loading and infusion to prevent filter clotting, and the dose range of heparin was 1500 to 6000 IU at each session of PMX-HP. According to our heparin dosing protocol [15], no premature clotting session (< 90 min) or substantial bleeding event was observed in this study. However, heparin might prevent microthrombosis in small vessels and protect glycocalyx from shedding by suppressing inflammation [26]. The referenced dose of heparin was 12000 IU/day for 7 days in a randomized trial for the treatment of sepsis [27]. Hence, additional studies are warranted to investigate the effect of heparin on microcirculation in patients with septic shock. Fourth, the SDF video microscope required an experienced operator to obtain good quality images, and sometimes the enrollment of patients in this study was limited due to unavailability of experienced operators. We suggest that further groups of microcirculation study are encouraged to train their research staff to obtain and analyze the microcirculation images according to the two consensus of assessment of sublingual microcirculation [16, 19], and communicating with experienced groups of microcirculation study is helpful.

In conclusion, this preliminary study revealed that PMX-HP treatment improved microcirculation but not clinical outcomes in patients with septic shock at a low risk of mortality. Nevertheless, larger multicenter trials are required to confirm the effect of PMX-HP treatment on microcirculation and clinical outcomes in patients with septic shock who have intermediate- and high-risks of mortality.

## Data Availability

The datasets generated and/or analyzed during the current study are not publicly available due to the regulation of the Research Ethics Committee of National Taiwan University Hospital but are available from the corresponding author on reasonable request.

## Abbreviations

APACHE: Acute physiology and chronic health evaluation
CPR: Cardiopulmonary resuscitation
EAA: Endotoxin activity assay
ICU: Intensive care units
MAP: Mean arterial pressure
MFI: Microvascular flow index
PaO_2_/FiO_2_: Arterial oxygen tension/fraction of inspired oxygen concentration
PMX-HP: Polymyxin B hemoperfusion
PPV: Proportion of perfused vessels
PVD: Perfused vessel density
SOFA: Sequential organ failure assessment
TVD: Total vessel density
